# Do Malawian women critically assess the quality of care? A qualitative study on women’s perceptions of perinatal care at a district hospital in Malawi

**DOI:** 10.1186/1742-4755-9-30

**Published:** 2012-11-16

**Authors:** Lily C Kumbani, Ellen Chirwa, Address Malata, Jon Øyvind Odland, Gunnar Bjune

**Affiliations:** 1 Institute of Health and Society, Department of Community Medicine, University of Oslo, Norway, P.O. Box 1130, Blindern, Oslo 0318, Norway; 2 Kamuzu College of Nursing, Blantyre Campus, P.O. Box 415, Blantyre, Malawi; 3 University of Tromsø, Tromsø N-9037, Norway

**Keywords:** Antenatal information, Perinatal care information, Perinatal period, Quality of care, Skilled birth attendant

## Abstract

**Background:**

Malawi has a high perinatal mortality rate of 40 deaths per 1,000 births. To promote neonatal health, the Government of Malawi has identified essential health care packages for improving maternal and neonatal health in health care facilities. However, regardless of the availability of health services, women’s perceptions of the care is important as it influences whether the women will or will not use the services. In Malawi 95% of pregnant women receive antenatal care from skilled attendants, but the number is reduced to 71% deliveries being conducted by skilled attendants. The objective of this study was to describe women’s perceptions on perinatal care among the women delivered at a district hospital.

**Methods:**

A descriptive study design with qualitative data collection and analysis methods. Data were collected through face-to-face in-depth interviews using semi-structured interview guides collecting information on women’s perceptions on perinatal care. A total of 14 in depth interviews were conducted with women delivering at Chiradzulu District Hospital from February to March 2011. The women were asked how they perceived the care they received from health workers during antepartum, intrapartum and postpartum. They were also asked about the information they received during provision of care. Data were manually analyzed using thematic analysis.

**Results:**

Two themes from the study were good care and unsatisfactory care. Subthemes under good care were: respect, confidentiality, privacy and normal delivery. Providers’ attitude, delay in providing care, inadequate care, and unavailability of delivery attendants were subthemes under unsatisfactory care.

**Conclusions:**

Although the results show that women wanted to be well received at health facilities, respected, treated with kindness, dignity and not shouted at, they were not critical of the care they received. The women did not know the quality of care to expect because they were not well informed. The women were not critical of the care they received because they were not aware of the standard of care. Instead they had low expectations. Health workers have a responsibility to inform women and their families about the care that women should expect. There is also a need for standardization of the antenatal information that is provided.

## Background

The perinatal mortality rate in Malawi is estimated to be 40 deaths per 1,000 births [1]. This is still high, although it is below the highest rate in Africa of 56 deaths per 1,000 births. In comparison with the developed countries the perinatal mortality is approximately 7 deaths per 1,000 births [2]. As a way of promoting neonatal health the Government of Malawi has identified essential health care packages for promoting maternal and neonatal health in health care facilities [3]. Provision of care in health facilities is based on the National Reproductive Health Standards [4]. The Reproductive Health standards are comprehensive and integrated. The following components specifically target perinatal care: focused antenatal care, normal labor and delivery, management of complications during labor and delivery and postnatal care of mother and neonate.

In addition to availability of health services, women’s perceptions of the care is important as it influences whether the women will use or not use the services. Hulton, Matthews and Stones (2007) assert that actual quality of care is theoretical for women if the experience deters some from returning for subsequent care [5]. Care given must result in women’s satisfaction for them to continue using the services. Quality of care “*involves providing a minimum level of care to all pregnant women and their newborn babies and a higher level of care to those who need it. This should be done while obtaining the best possible medical outcome, and while providing care that satisfies women and their families and their care providers”* (Pittrof, Campbell & Filippi (2002 p.278) [6]. Similarly, in Malawi quality of care focuses on mutual satisfaction of both the users and providers. According to National Reproductive Health Service Delivery guidelines, quality is “doing the best with the resources available for the mutual satisfaction of the provider, the recipient (in this case the client/patient) and community at large” (Ministry of Health, 2007 p.1) [7]. Quality of care is in this study focused on both the provision of care as well as how clients perceived the care. This was based on Hulton, Matthews and Stones (2000) who looked at quality not only as provision of services, but as also women’s actual experiences of that care [8].

In Malawi 95% of pregnant women receive antenatal care from a skilled attendant (doctor, clinical officer, nurse, or midwife) according to the Malawi Demographic Health Survey 2010. However, the percentage is reduced to 71% for deliveries with 84% in urban areas compared to 69% in the rural areas [1]. This is an improvement from 2004 where assistance by a skilled attendant at delivery was at 57% [9]. Malawi has a larger rural population which comprises 84.7% of the population (13,077,160) compared to 15.3% urban population [1]. Consequently, there is still a need to ensure that more pregnant women, especially from the rural areas, deliver with skilled attendants to reduce perinatal deaths. Providing access to skilled attendance at every delivery and early recognition and timely management of obstetric complications through emergency obstetric care (EmOC) are crucial in reducing perinatal mortality [10]. However, for this to happen, pregnant women need to deliver in health facilities in places where skilled attendants are not available to assist in home deliveries. A study in Timor-Leste observed that many women continued to deliver at home although they had easy access to new birth facilities following a national policy promoting hospital deliveries [11]. Pregnant women are more likely to deliver in health facilities if they are satisfied with the care that they receive when they go there. Poor quality of care may lead to underutilization of services [12,13]. In a study conducted in rural Zimbabwe, poor quality of services and negative attitudes of health workers deterred pregnant women from utilizing health services [14]. Similarly, non-utilization of health facilities for delivery in Nigeria was related to unsatisfactory services at health facility for more than half of the participants, and unfriendly attitudes of staff for the majority [15].

Conversely, satisfaction with care does not essentially mean that quality is good, as it may indicate low or no expectations [16]. Women need to be well informed about the care they should expect. Information provided to pregnant women covers issues from pregnancy, labor and delivery and postnatal periods. Women depend on health workers to give them information on health issues [17]. There is some evidence that high quality patient- provider information during pregnancy will result in favorable pregnancy outcomes for the women [18]. This is probably because women who are well informed are able to demand and receive appropriate care. In a study done in Australia, midwives and nurses were believed to be important sources of information and their advice was most likely to be followed [19]. Antenatal information is useful for women as it helps them prepare for delivery [20]. One study has demonstrated that antenatal information contributed to maternal satisfaction with labor management and parenting experiences [21].

In Malawi, as in other countries, attending antenatal care at health facilities provides an opportunity to large numbers of pregnant women to receive correct information about pregnancy, labor, delivery, and postnatal care. Malawi adapted WHO essential guidelines of focused antenatal care provision within four visits. A set of interventions are provided at each visit of the four visits to ensure normal pregnancy, prevent complications and facilitate early identification and treatment of complications [22]. Activities include client education and specific advice and counseling as needed that is given throughout the four visits. The areas include: process of pregnancy and possible complications, danger signs in pregnancy, labor and delivery, postnatal and in the neonate, importance of colostrum, early initiation of breastfeeding, birth preparedness with focus on skilled attendant, signs and symptoms of onset of labor, and postnatal care [7]. Information on birth preparedness comprises male involvement, skilled attendance at birth, choice of place of delivery, arrangements for transportation, source of funds, decision making by the family including a labor companion during labor and childbirth, items needed for a clean and safe delivery, danger signs and what to do if they occur [23]. The assumption is that provision of normal antenatal care is uniform in all health facilities as it is based on defined guidelines.

If health workers in Malawi follow the given antenatal care matrix, then appropriate information on what to expect during perinatal care, including labor and delivery is provided to women. The women may also have other sources of information that might influence utilization of health services. This is mostly through radios in the rural areas that broadcast messages on safe motherhood and health facility deliveries. The latest demographic health survey found that 57% of women listened to ‘Safe Motherhood’ programs, although it was lower (55.3%) for rural women [1]. Lack of appropriate information for women to be aware of what to expect makes them complacent about the care they receive. The women have low expectations as they do not know [24].

The Malawi government’s policy is that women should use health facilities for perinatal care. That is antenatal care, delivery and postnatal care for the mothers and babies. Information on women’s perceptions of perinatal care is scarce in Malawi. Understanding women’s perception of care can inform the provision of quality care that is acceptable to women. This study was conducted to explore women’s views of perinatal care. The objective of the study was to describe perceptions of perinatal care among women who delivered at a district hospital in Malawi.

## Methods

### Study design

The design was a descriptive qualitative study using a semi structured interview guide. Face-to-face in-depth interviews were conducted. Participants who delivered from February to March 2011 were asked to describe their perceptions of perinatal care. The women were asked how they perceived the care they received during antepartum, intrapartum and postpartum. They were also asked about the information they received during provision of care. The interviews were conducted in the local language, Chichewa, using a semi structured interview guide. Throughout the interviews, follow-up questions using probes were asked in order to acquire a deeper understanding when an explanation was unclear. The interviews lasted on average 45 minutes. All the interviews were recorded, translated and transcribed verbatim in English.

### Setting

The study was conducted in the Southern region of Malawi at the Chiradzulu District Hospital. Chiradzulu district has a total population of 288,546 people, with 153,200 women. Twenty three percent are women of the child bearing age of 15–49 [25]. Two districts, Zomba and Chiradzulu, had the highest neonatal mortality in the Southern Region at 48 and 47/ 1000 live births respectively, compared to the national average of 33/ 1000 live births in 2006 [26]. Chiradzulu district is a rural area serviced by a district hospital while Zomba is more urban with a central hospital. Chiradzulu District Hospital is a secondary level facility with 11 health centers that offer primary level care. Ten of these 11 health centers provide maternity services (antenatal, labor and delivery and postnatal care) and refer women with complications during perinatal period to Chiradzulu District Hospital. Data were collected from women who delivered at Chiradzulu District Hospital and attended antenatal care at either the district hospital or any other facility.

### Participants’ recruitment and data collection

Mothers who delivered normally, were well, and had consented to participate in the study were selected using purposive sampling. Purposive sampling is a non-probability sampling method that facilitated that various groups of women based on age and parity were represented [27]. The mothers were selected based on the fact that they had delivered and were able to provide information on perinatal care. The first author provided information to and recruited potential participants that met the defined criteria. All women who were approached and asked to participate in the study accepted. Mothers who deliver normally without complications in themselves or their babies are discharged within 24 hours. The mothers who were interviewed stayed in postnatal ward after delivery for periods ranging from less than a day to seven days before they were discharged. The interviews were consequently done within 24 hours of delivery to seven days after delivery. All interviews with the mothers were done after they were discharged to make certain that they would express themselves freely. The first author conducted all the interviews. An empty room was identified in the postnatal ward for the interviews to ensure privacy and comfort of the participants. Interviews were conducted until saturation of data was reached. There was no additional new information later. Instead, there was repetition and confirmation of already collected data [27]. A total of 14 mothers from the district hospital were interviewed.

### Data analysis

ATLAS. Ti version 6.2 computer software program was used to code the transcripts and for storage of the data. The Statistical Package for Social sciences (SPSS) version 18.0 (SPSS Inc. Oslo, Norway) was used for data entry and descriptive analysis of the participants’ demographic data. Qualitative analysis focused on participants’ perception of the care they received during antepartum, intrapartum and postpartum; what they liked about the care they received and what the problems or constraints were. All recorded interviews were transcribed verbatim in full. The analysis focused on developing coding categories where narrative information was organized according to emerging themes using thematic analysis [28]. Coding of the data was done without fitting it into a pre-existing coding frame. The data were read and re-read to identify themes that were related to quality of care.

### Ethical consideration

The Norway Regional Committee for Medical Research Ethics granted approval for the study as well as the College of Medicine Research and Ethics Committee (COMREC) in Malawi. A written permission was obtained from the District Health Officer (DHO) of Chiradzulu District Hospital to conduct the study.

Participants that agreed to participate in the study signed an informed consent form or gave a thumbprint if they were illiterate. Participation in the study was voluntary and participants were assured that anonymity would be observed at all times. Confidentiality of participants was maintained by using numbers on both the recorded interviews and transcripts.

### Limitations

The study might suffer a bias because the participants were interviewed on the premises of the district hospital, where they had received care. Although the interviews were conducted after the participants were discharged, this may have influenced some of their responses. The first author who collected the data is a health professional but does not work at the study site. Participants’ knowledge of her professional status may have influenced some of their responses. To reduce reflexivity the interviewer was aware of possible prejudices. She adjusted in order to collect significant data that was undistorted by personal prejudice [29]. Some of these women were multiparas and although the questions asked about the last pregnancy experiences they may have included experiences from previous pregnancies resulting in some recall bias.

## Findings

### Demographic data

The majority, (n = 11) of the participants had primary education and one had no education. The average age of the women was 30 years, (with a range of 15 years and 44 years). The number of antenatal visits ranged from one visit to more than five visits with an average of 3.4 visists. The parity of the participants ranged from one to eleven, and average was 4.7. Half of women who delivered at hospital had given birth at least five times. Half of the women stayed at the health facility for a day or less days after delivery. All participants had HIV status checked and the majority were tested for syphilis. Four women were HIV positive while one had syphilis out of 11 women tested for syphilis (Table 1). The participant who had syphilis was treated. The majority, (n = 13), were married. Three participants received antenatal care from the district hospital because it was their nearest health facility while the rest it was from various health centers.

**Table 1:** Demographic characteristics of participants (n = 14)

**Characteristic**	**Number**
**Education level**	
No education	1
Primary	11
Secondary	2
**Age group**	
15-19	3
20-24	3
25-34	1
35-49	7
**Number of ANC visits**	
1	1
2	3
3	3
4	3
≥5	4
**ANC screening**	
**HIV testing:**	14
Positive	4
Negative	10
**Syphilis:**	11
Positive	1
Negative	10
**Parity**	
1	4
2-4	3
≥5	7
**Duration in hospital after delivery (day/s)**	
≤1	7
2	2
3	0
4	3
≥5	2

### Perceptions of perinatal care

Using thematic inductive analysis [28] good care and unsatisfactory care were major themes that were identified. Subthemes under good care were respect, confidentiality, privacy and normal delivery. Providers’ attitudes, delay in providing care, inadequate care, and unavailability of skilled attendant were subthemes under unsatisfactory care. Some of the participants did not know and were not able to describe their perceptions about care that was provided to them. The themes and subthemes are described in detail below.

### Antenatal care

#### Good care

Participants described their perceptions and experiences of antenatal care they received in various health facilities including the district hospital. Participants perceived good care when being respected, not handled with rudeness or being shouted at, being given medicines and mosquito nets. Participants felt the care was good because they received medicines; some mentioned mosquito nets that protected them from frequent attacks of malaria. Although antenatal care comprises several activities, the participants only highlighted these two aspects of receiving medication and mosquito nets.

#### Good reception and respect

Nearly all participants described the care they received as good or adequate. The participants narrations indicated that the way the participants were welcomed at the clinic greatly influenced how they felt about the care they received. The women valued most ‘being welcomed and respected by health care workers’ which seemed to be important to them as explained by participant #12.

“I see that I was well received, they received us and did not shout at us”.

Similarly, participant # 7 explained that:

“They received me well without being disrespectful. Others are rude to you, this and that but in my case, I was received well as a fellow woman”.

### Provision of information

Participants reported that they were given information on various health issues during group health education. The topics that women recalled were on health promotion, prevention of malaria, prevention of mother to child transmission of HIV (PMTCT), breast feeding options, birth preparedness, danger signs in pregnancy and what to do if they experience complications (Table 2). The women mentioned severe headache, severe abdominal pains, vaginal bleeding and draining liquor as danger signs in pregnancy. The mothers also included other diseases like malaria. Most participants, including those who had forgotten or did not know any danger sign, knew they had to go to a health facility if they had any problem.

**Table 2:** Antenatal health education areas mentioned by participants

**Topic**	**Area**
Health Promotion	Eat nutritious diet
	Sleep in a mosquito net
	Go to antenatal clinic as scheduled
	Go to antenatal clinic to be assessed
	Reduce workload
Prevention of mother to child transmission of HIV (PMTCT)	Being faithful to each other
	Use of condoms
	Avoid extra marital affairs
	Use of antiretroviral therapy (ART) for the mother
	Giving medicine to the baby after delivery- antiretroviral syrup
	Getting tested
Feeding Options	Exclusive breastfeeding up to six months
	Frequent breastfeeding of the baby
	Breastfeed up to six months then stop if HIV positive, or do not breastfeed at all.
	Breast milk is important, has everything
Danger Signs	Severe headache, severe abdominal pains, malaria, HIV, vaginal bleeding, draining liquor, side effects of ART, nose bleeding
What to do when Danger signs occur	Go to the hospital quickly
	Go to the hospital quickly to prevent complications
	Go to the hospital
Birth Preparedness	Signs of labor
	Have cloths for the baby and mother
	Buy necessary things: basin, cloths, razor blade, thread, plastic paper, perineal pads.
	Go to the hospital quickly when labor starts
	Go to the hospital to be assisted
	Go to the hospital, do not deliver at home; with traditional birth attendants

Nevertheless, the participants did not associate provision of information to care. Only one participant mentioned provision of information as an aspect of good care. Although she was not the only young primipara, this participant was given information about onset of labor and hospital delivery.

“They gave me advice and told me that when I see these things then it is a sign of labor and I should go to the hospital quickly. This was good”. Participant # 3

### Unsatisfactory care

Some of the same participants who stated antenatal care was good were also able to point out areas that they were unhappy with during provision of care. These were older multiparas. The participants described what they did not like in terms of how the health workers communicated and provided care to them.

### Provision of information

There was minimal communication during provision of care although participants did not mention this as a concern. Participants stated that health workers were not providing feedback or information to them. Participants explained further that they were not told anything but were just examined, given some medicine and given date for the next visit.

“All they said was we are done after examining you. The results they keep to themselves after examining you. You are just told to go when they are done.” Participant # 10

### Providers’ attitudes

In Malawi, health workers’ attitudes are a great challenge in the provision of health care especially during labor and delivery. Some health workers were accused of providing care in a discriminatory manner. For instance at health facilities that use an integrated approach, care was prioritized to family planning clients instead of on first come first served basis. A participant reported that she stayed at the antenatal clinic for a long time because the health workers examined her after finishing examining family planning clients. Participant # 11 explained as follows:

*“Checking of weights was done in good time but physical examination was done late. They started with family planning clients before examining pregnant women”*.

### Labor and delivery care

#### Good care

Participants were describing their perceptions and experiences of labor and delivery care they received at the district hospital. Some of the participants that were referred to the facility also included aspects of care at the referring facility. Participants rated intrapartum care as good when; reception was good, they were respected, their privacy and confidentiality were maintained, and when they delivered normally. A minority of the participants described technical aspects of care that was provided during labor and delivery. A participant, who was referred from a health centre while in labor, stated that care was adequate at the health centre because she was examined frequently and commenced an intravenous infusion. She further reported that care at the hospital was even better because they commenced another drip once the first one from the health centre finished. Another participant # 11 described her care as:

“When I delivered I was given a cloth to use as a sanitary pad. The nurse was not disgusted that I was soiled, she wiped me clean. The cloth that was underneath was removed. Therefore I see that I was properly cared for”.

#### Good reception and respect

Nearly all of the participants reported they were well received in labor ward. The reasons given were they were not shouted at, neither spoken to harshly, or with anger. They were spoken to with gentleness, were respected and quickly assisted and delivered when they came distressed in labor. This experience was shared by participant #13:

*“When I went in I was told to lie on a bed. A midwife told me to take my paper (plastic paper) and spread it on the bed and then to put my cloth on top”.* …*Some shout but they received me well”.*

Respect was associated with how participants were received upon admission to the labor ward and how health workers communicated. Other services that health workers provided to clients were also rated as respect. Participant # 1 shared as follows:

“When I was in labor, a nurse brought me porridge for me to have energy during delivery of the baby. I saw that I was respected”.

### Confidentiality and privacy

A minority of the participants reported on confidentiality in provision of care. Participants described confidentiality as health workers keeping secrets about delivery issues. Participant #2 explained how confidentiality was maintained regarding her experience during labor and delivery.

“It was a man who delivered me but he did not say anything. He just helped me as a doctor. If he was someone else he would be saying I am the one who delivered her, that person this and that. In that way I see that, the doctor kept confidentiality. He did not say anything to anyone. He was just doing his job”.

All participants mentioned that privacy was maintained but said so only after probing. The participants explained that they were screened and spoken to quietly and properly to maintain privacy.

*“The curtains were drawn so that people passing by would not see what was happening inside”.* Participant # 9"

### Normal delivery

Participants also perceived care as being good when they delivered normally without any interventions. Participants linked good care to normal spontaneous delivery and fewer than half expressed it clearly. Participant # 7, whose baby had fetal distress, shared her experience as follows:

“The care was good and I am thankful. If I was home it would not have ended well, the baby or myself would have died the way things were. I have been helped a lot at the hospital. I am well and the baby is well too”.

Some participants who experienced a negative birth outcome also perceived the care they received as good despite the outcome. Participant # 8 delivered a macerated still birth and said:

“I am thankful; they (health workers) helped me. I have seen what I was waiting for. I was coming here for the doctors because on my own I would not have managed”.

A minority of the participants were not able to give any information about labor and delivery care they received. The participants reported that a health worker was the one who knew what to do. A participant stated that there was nothing else she would have liked to be done as she did not know what happens. Care was connected to assistance during delivery of the baby for some participants. A participant who could not elaborate on the type of care shared as follows:

*“No nothing, provided you deliver well. There is no need to add anything else”.* Participant # 12

### Unsatisfactory care

Some of the same participants who stated good aspects of intrapartum care also highlighted care that made them unhappy during provision of care. Both multiparas and primiparas shared some of the things they did not like. They are described in the following sections.

### Providers’ attitudes

The participants expressed concerns about poor attitudes of health workers. Participants wanted to be communicated to appropriately by health workers and not to be belittled or degraded. A participant, who was referred from a health centre, was distressed on admission to labor ward. She said a female midwife talked to her as if she was talking to a child. She even told her not to complain. In the course of this participant’s labor when she called out to the midwife, she was ridiculed. She reported a midwife shouted back in response:

*“The female nurse when I called she answered that I was not near in a loud voice. She was on another side helping someone else”.* Participant # 6

Participants narrated different situations in which the health workers treated them harshly. Participant # 10 who was referred described her reception at the district hospital as follows:

“When I came here they asked why I had come. They told me that I would be sent back to deliver at the health centre where I came from. They said that there were rules these days that states that women should not come and deliver at a district hospital without valid reasons. They said that if I did not answer their questions properly I would be sent back to deliver at my health centre. So I explained why I left the health centre and came here”.

Participants reported that they wanted to be treated well and talked to us with kindness. They expected health workers to show kindness towards their clients in their work. Some participants explained that women sometimes are shouted at during labor because they provoke the health workers. Such situations arise when the women do not take health workers’ instructions. Participant # 8 narrated as follows:

“You know with labor pains you cannot say I know everything because I am old and I have been giving birth since a long time back. Doctors tell you to “do this”, but because of the way you are feeling you fail to do as you are told. As a way of wanting to save you, they may shout at you depending on how you are behaving. Sometimes we provoke the doctors because of how we behave”.

However, the same participant later said:

“.. If somebody tells you something while shouting, you feel threatened. They (health workers) should talk to us nicely and tell us information kindly”.

### Delay in providing care

Participants did not like not being attended to as soon as they reported to the labor ward. Some participants, who were in labor on admission waited to be assessed. They were told to go and lie on the bed and a nurse midwife would find them there. A participant reported that she delivered within 30 minutes of admission but, was made to sit and wait on a chair while a bed was being cleaned for her. She felt this was not good as she was in severe pain. Participant # 10 who waited to be assessed on admission shared her experience:

*“I was told to go and lie on the bed and she (health worker) would find me. ….My eyes were closed as I was in pain. After some time had passed, she came on her own (to assess me)”*.

The participants explained that when they go to a health facility, the health workers should attend to them quickly.

*“The health workers should assess us quickly and inform us immediately. The health workers know because they were educated so they know how things are; we are pregnant but we are ignorant of what we are carrying”.* Participant # 8

### Inadequate care

The participants’ narrations indicated that the women viewed the care that was provided as being not comprehensive enough. A participant reported that she did not receive any care apart from assistance during the delivery itself.

Lack of pain relief bothered some participants. Participants described lack of pain management as a problem for them. The participants expected that the health workers would explain to them what to do when in labor and feeling pain. Participant # 3 explained as follows:

“I expected that the health workers would be supportive. They should tell us what to do when you experience labor pains.”

Another, a primipara shared the following:

*“I was in a lot of pain and I was thinking I should go for an operation but they told me that I would deliver, they did not give me an injection to reduce the pain, so the pain was unbearable”.* (Participant # 6)

### Unavailability of delivery attendant

Participants desired midwives to be with them and provide relevant care during labor and delivery. A participant, who delivered on her own at the health facility, said she was pushing alone but called when she felt the baby, was coming. She was afraid she might harm or kill the baby.

*“I would have loved if a midwife was there when I was delivering, to ensure safe delivery of the baby”.* Participant # 4

Another participant explained as follows:

*“I would experience pain then it would stop. This (*the pain*) was happening at a time when a nurse should have been close to help but at that time she was not there”.* Participant # 10

### Postnatal care

#### Good care

Participants described good postnatal care when they were taken from the labor ward to postnatal ward in a wheel chair, given medicines and a bed on which to sleep. Participants explained that they were exhausted, some felt dizzy or legs were numb and had problems to walk from the labor ward to postnatal ward. Participant # 7 shared that:

“From the Labor ward I was taken in a wheel chair (to a postnatal ward) where I was given a place. Later on, a doctor called and asked me to take the baby to him. The doctor gave the baby some medicine (antiretroviral syrup) and he told me that I would be called again the next day. Today I was called again.”

#### Provision of medicines

Fewer than half of the participants appreciated that they were given medicines. The participants described provision of medicine to themselves for after pains, or their babies who were sick, and antiretroviral syrup as prophylaxis. One of the participants related good care to the medical treatment she received:

*“I was given medicines and an intravenous infusion drip. They found me with malaria in labor ward. They were caring because of just the medicine… since what you want is to get well”* Participant #5

Similarly, the mother of a baby whose fever was detected during assessment prior to discharge and was put on treatment for sepsis was happy with the care she received.

*“I went for check up where they saw the baby had developed fever. You should stay here for now. The baby will be receiving treatment. He will receive four injections per day. I was given good care”.* Participant # 2

#### Provision of information

Participants were given information about breast feeding, baby care, danger signs and postnatal checks after delivery (Table 3). About half stated they were not given any information on danger signs and none mentioned poor suckling or feeding. Mothers go with their babies to a nearest health facility for postnatal check up at 1 week and six weeks. The majority of the participants were informed to go for postnatal check up at one week but only a minority of them were told to go again at 6 weeks. A minority of the participants were told about the significance of postnatal checks. This was for the babies to be reviewed; two of the babies were getting antiretroviral syrup. However, the mothers did not relate provision of postnatal information to quality of care.

**Table 3:** Postnatal health education areas mentioned by participants

**Topic**	**Area**
Baby care	Frequent breastfeeding
	Exclusive breastfeeding up to six months
	Warmth
	Keep the baby on the chest KMC*
	Check cord for bleeding
	Cord care
	Bathing the baby
Danger signs	Fever
	Baby just crying
	Loss of consciousness
	Epistaxis
	Pus from the cord
What to do when Danger signs occur	Take the baby to the hospital

KMC* - Kangaroo Mother Care for a preterm baby.

#### Unsatisfactory care

Although participants mentioned positive aspects of the care they received, they also pointed out few things they did not like. This was from both a primipara and multiparas.

#### Inadequate care

Participants stated that after delivery they were escorted to postnatal ward where they slept without any assessment.

*“I was taken on a wheel chair to come here. When I arrived here the nurse did not receive me but she did the next morning. When I came here I slept”.* Participant # 2

However, this participant did not see this lack of assessment as a problem:

“It is not a problem, maybe it is how they work. Maybe the time for them to work was over”.

A minority of the participants stated they were in agony from after pains and only ate porridge after delivery and nothing else until the following day. A participant reported to a nurse on admission to postnatal ward and got medicine, while another did not report and did not get any medicine. Yet another participant reported she was given medicine but she did not know what they were and why:

*“I was given medicine.* … *I do not know whether it was bactrim or panadol*….*They just asked who delivered today and gave us the medicine. I do not know what it was for. I just received since I am in hospital”.* Participant # 4

### Definition of terms

Perinatal period: from 28 completed weeks of gestation to seven completed days after birth.

Quality of care: focuses on both the provision of care as well, as how clients perceive the care.

Skilled birth attendant: a skilled attendant is an accredited health professional such as a midwife, doctor or nurse - who has been educated and trained to proficiency in the skills needed to manage normal (uncomplicated) pregnancies, childbirth and the immediate postnatal period, and in the identification, management and referral of complications in women and newborns.

## Discussion

Skilled attendance at delivery is essential in reducing perinatal morbidity and mortality because when complications arise they are effectively managed. Pregnant women will deliver in health facilities if they are satisfied with the care that they receive. However, for women to be really satisfied they must be well informed and know what to expect. This will then provide a basis for possible scrutiny. The findings in this study have shown that participants did not know what to expect. However, the participants viewed being respected, good reception, being assessed quickly and informed about the findings, confidentiality and privacy as being good quality of care.

The participants focused on the interaction with the health workers, which is an important aspect of quality of care. Shafiei, Small, & McLachlan, (2012) demonstrated that interactions with health workers were particularly important in the satisfaction or dissatisfaction of the care women received [30]. Humane aspect of care provision is a very important part of the quality of care. Bazant & Koenig, (2009) found that health care provider empathy had a great effect on the women’s satisfaction with care [31]. Similar findings were that women wanted to be treated with tender loving care during childbirth as they go through a difficult time and need the moral support of health workers [32]. The women wanted a caring attitude and empathy from the health workers. The participants in this study who were satisfied with the care they received did not want much from health workers. They wanted to be received well. Participants highlighted being assessed quickly and being informed about the findings, receiving medications, that health workers were not harsh, did not shout and spoke with gentleness, treated them with respect, maintained confidentiality and privacy as good experiences of care.

Participants who experienced complications but delivered normally were satisfied with the care they received. The presence of complications, but with a good outcome, may affect how these participants perceived the care they received. Pittrof, Campbell, & Filippi, (2002) assert users to be satisfied with care if they have a good outcome [6]. These participants may simply be very relieved [31]. Participants in this study showed that a normal delivery overrides complaints.

A participant in labor who was told to put a plastic paper and cloth on the bed by herself before lying down was satisfied with the way she was received. This was unacceptable, as beds are made by health workers. In addition, the women have to be helped to climb up on to beds to lie down. Another participant even justified being shouted at by health workers. Possibly the participants felt they should tolerate the health workers’ behavior in order to be assisted. This is consistent with Yakong, Rush, Bassett-Smith, Bottorff, & Robinson`s (2010) findings where women accepted disrespect, intimidation and scolding they received order to obtain care [33]. Similarly, Delvaux et al. (2007) observed that most women were satisfied with the care they received during and after labor despite low technical quality and unfavorable personal support given to women [34].

The participants in this study did not comment much on the technical skills of health workers. It is perhaps because the participants did not know what to expect. Thus, they were grateful for whatever was done for them. This is different from Goberna-Tricas et al. (2011) study findings in Spain where women clearly distinguished between technical and interpersonal skills and commented on both [32]. The women were happy with the technology but also wanted humane care from health workers just like the participants in this study. It is important to note that the majority of the participants in this study had only primary level education while education level was not reported for the participants in Spain.

The narrations of the participants demonstrate that they received poor quality of care. Health workers attended to antenatal clients last after finishing family planning clients. They were not providing the necessary information to the women, lack of support and pain relief for women in labor, a participant who delivered on her own and asking the women to justify their referral to the hospital. It was improper to ask the referred women to explain the reason for referral. The women come with documented information from health workers at the referring health facility. In addition, this is a referral hospital that receives pregnant women with complications throughout the perinatal period from its health centers. Although the National Reproductive Health Standards guide the provision of care in health facilities, none of the standards were available in labor ward and postnatal wards during data collection. However, I was informed that they are kept in Maternal and Child Health Department. This causes doubt if health workers actually use the guidelines in the provision of care.

The women may not be aware of the type of care they are supposed to get because of their limited understanding. Muula (2005) found that nearly all (96%) participants did not know about health rights and it was also stated that patients seemed prepared to be shouted at [35]. A study done in an urban setting, at a central hospital in Malawi found that about half (51%) had ever heard about patients’ right and the most (57.4%) mentioned the right of a patient to have considerate and respectful care [36]. No studies were found on patients’ rights in a rural setting in Malawi. There was no information on patients’ rights given to clients at the hospital during data collection period. It was only late in the year 2011 that health facilities had Charter of Patients and Health Workers’ Rights and Responsibilities posters and fliers. The Ministry of Health with assistance from Deutsche Gesellschaft für Internationale Zusammenarbeit (GIZ), distributed the posters and fliers from November to December 2011. The participants therefore might have lacked information on patients’ rights that would help them to demand for quality care. They therefore accepted whatever care that was provided. This emphasizes the need for health workers in Malawi to inform women and their families on what to expect not only in pregnancy but also during labor, delivery and post delivery.

The observation that clients were not given adequate information during pregnancy is similar to findings by other studies in Malawi [37,38]. This may be one of the reasons the participants were not able to assess the type of care that was provided. The participants were not told and they had no basis for comparison. Therefore, these participants did not complain about the care they received because they did not know what to expect. Both the participants that were happy with the care they received and those that could not express their views were possibly satisfied. Their expectations were low because they have never experienced any other standard of care [16,24]. Another reason for not complaining could be the timing of the interviews. The participants might not be critical because they were happy, they had just delivered. However, there were no differences in the participant’s responses based on the time of interviews, within 24 hours to seven days after delivery. Conversely, studies done elsewhere having similar interview times, such as immediately after delivery and the other within 48 hours after delivery [39,40], and exit interviews on discharge [36,41,42] showed that the participants were critical of the care they received. There is a need for antenatal information to be standardized in the guidelines for easy implementation. Emphasis should be made for health workers to give feedback and information to clients after assessment based on clients’ unique information needs. Proper supervision, monitoring and capacity building of health workers are necessary to ensure that women and their families are provided with appropriate information.

The participants had poor knowledge of danger signs that occur in newborn babies. When mothers are well informed they are more likely to take their babies to health facilities when problems occur [43-45]. There is a need to strengthened information on newborn care during antenatal care and emphasized again during postnatal care. It is essential that health workers correctly inform mothers about these danger signs. This is vital as most mothers are discharged early within 24 hours after a normal delivery.

The current Prevention of mother to child transmission of HIV guidelines advocate for early initiation of antiretrovial therapy (ART), starting at 14 weeks gestation and continue through labor and breastfeeding. This is to slow progression, increase survival and reduce transmission of HIV to the baby. The guidelines also recommend initiating ART after a positive HIV result of the mother during labor or after delivery [46]. However, majority of the participants that tested HIV negative in pregnancy were not checked again after delivery. The participants were given no information that they should be tested again. This is a missed opportunity as some of the participants could be infected with HIV at this time and may transmit to their babies during breastfeeding. An HIV test is recommended for an individual who tested negative more than 3 months ago [46]. It is therefore important to use this chance to repeat an HIV test while the mothers are still at the health facility as well as providing required information.

Participants pointed out areas that they were unhappy with during provision of care. The participants identified providers’ attitude, delay in providing care, inadequate care, and unavailability of delivery attendant as negative experiences of care.

Attitudes of health workers are an important aspect of care. This is because women value how they are treated when they present themselves at health facilities for care. The participants in this study did not like the behavior of health workers who were rude or shouted at them. Issues of poor attitudes of health workers found in this study are reflected in other studies in Malawi [24,47]. This observation is also consistent with Small, Yelland, Lumley, Brown, & Liamputtong`s (2002) findings where immigrant women met unsympathetic, uncaring, or rude staff that distressed them [48]. Similarly, Yakong et al., (2010) showed that women were scolded, threatened with treatment withdrawal or denial if they did not comply with nurses’ instructions [33]. They were treated ‘like children’, ignored, and disrespected. If women are not well treated in health facilities, they will continue going for antenatal care but not return to deliver in health facilities. Women were reluctant to seek care in subsequent pregnancies in a study in Timor-Leste because of poor attitudes of health workers, who easily got angry, left women alone or shouted at them [11]. Likewise, health workers who used abusive language, showed no tolerance and had hostile behaviors deterred women from using health facilities for deliveries in Malawi, rural Tanzania and Ghana [47,49,50].

Afghan women’s waiting time at antenatal visits and in postnatal ward negatively impacted on their experiences of care [30]. These women were not in labor, what more with women who are experiencing labor pains. The women when they come in labor need to be assessed as soon as possible to enable formulation of an individualized plan of care. Inadequate staffing and high workload may influence nurse midwives client-unfriendly behavior [51]. Nevertheless, delayed assessment of woman in labor may result in no assessments until the woman delivers. One participant in this study delivered alone. The 2010 Malawi Demographic Health Survey found that 3% of deliveries in health facilities were self-deliveries, attended by no one [1]. Seljeskog et al., (2006) study in Mangochi, Malawi established that women in labor waited very long to be examined and that some even delivered without any assistance, supervision or attention at all [24]. Foster et al., (2010) found that prolonged waiting period of pregnant women at the health facility was related to fear of being ignored or neglected [52]. It is important that women be quickly assessed on admission to the labor ward for appropriate management.

Pain during labor is a normal physiological process. Sadly, most health workers feel women must endure the pain. A study in Benin found that midwives expected women to be stoic to labor pains and complaints were treated with mockery and humiliation [53]. Women in labor know that pain management is an essential component of providing quality care to women in labor. Goberna-Tricas et al., (2011) showed that women thought the experience of pain was unnecessary and it was sensible to avoid the pain [32]. Similarly in another study, the majority, of the women stated they would want pain relief in labor [54]. Small et al., (2002) asserted that how women rated intrapartum care was dependent on how the women felt about what was done to relieve pain [48]. Health workers are expected to provide non-pharmaceutical and pharmaceutical pain relief during labor [4]. However, most of the times, drugs are in short supply and pain relief may not be a priority among health workers. Health workers’ convictions about labor may also affect their use of both non-pharmaceutical and pharmaceutical pain relief measures.

Women want to have a nurse midwife available when they are in labor and during delivery. Women’s perceptions of staff as being unhelpful and uncaring by not offering comfort or being absent contributed vastly to women’s dissatisfaction with their care in labor [48]. Wild et al., (2010) found that when women were left alone they were reluctant to seek care in subsequent pregnancies [11]. Gao et al. (2010) reported that women preferred to deliver at home because nobody looked after them in the hospital [55]. A woman, who delivered alone because it was between shifts, was afraid to go to the hospital for subsequent pregnancy after that. Similarly, in Malawi women delivering alone prevented them from going to deliver in health facilities [47].

According to the 2010, Malawi Demographic Health Survey skilled attendants delivered 71% of women who went to health facilities for deliveries [1]. Despite this, what is significant is the quality of care provided. Poor quality of care was related to 49% of maternal deaths that occurred in the Southern region of Malawi [56]. Other studies have also established that poor quality of care is provided in health facilities [24,37]. Health workers perceived that they provided substandard care according to a study looking at provision of emergency obstetric care at a district hospital [57]. Furthermore, a quality gap was evident in provision of newborn care services [58]. It is essential for health facilities to provide quality care because it is effective in preventing and managing complications and sustains demand for health care [59]. It is not enough to ask women to go and deliver in health facilities and not guarantee that they are given the necessary care during labor and delivery. In addition to availability of skilled attendance at delivery, health workers must provide quality client friendly services throughout the continuum of care.

Thorough assessment of women and their neonates when they are admitted in postnatal ward is important to identify any problems that may have arisen. Neonates are vulnerable especially during the first 24 hours after delivery [60]. Lawn, Cousens, & Zupan, (2005) analysis demonstrated that out of perinatal deaths 25% to 45% occur within the first 24 hours of life [61]. It is therefore necessary that neonates be assessed when they go to the postnatal ward. However, women and neonates without problems are not usually assessed in postnatal ward until it is time for discharge. This is despite that guidelines in Malawi specify that daily assessments should be done on neonates in postnatal ward [23]. It is necessary to enforce these guidelines that the neonates should be assessed on admission to postnatal ward or whilst in the ward and not only on discharge.

## Conclusion

The women in our study were able to describe what they perceived as good care. The women also described care that was not satisfactory for them. However, the women did not know the quality of care to expect, as they were not well informed. Women’s perception of care is important as it contributes to women’s satisfaction with services. This is crucial in promoting that pregnant women deliver in health facilities with skilled attendants to promote wellbeing of the neonates. There is also need to ensure that health workers are aware of how their attitudes affect women’s health care seeking behaviors. Health workers should not take advantage of women’s ignorance to provide poor quality of care. Health workers should show empathy and provide care that is acceptable and suitable to all women based on set guidelines. Health workers have a responsibility to inform pregnant women and communities about perinatal care. Standardized antenatal information is necessary to ensure provision of appropriate information to clients. Ultimately, this may enable clients to identify and demand provision of care that is satisfactory and appropriate for them.

## Competing interests

The authors declare that they have no competing interests.

## Authors’ contributions

LCK conceptualized the study, collected, analyzed data and drafted the manuscript. JØO is the Principal supervisor of the study. JØO, EM and AM have critically reviewed drafts, edited and provided important intellectual content. GB has reviewed the drafts critically, edited and given important input to the content and discussion. All authors read and approved the final manuscript.

## Authors’ information

LCK is currently a PhD student at the University of Oslo. She holds a M.Sc. degree in Nursing and is a Senior Lecturer in Midwifery at Kamuzu College of Nursing, Blantyre Campus in Malawi.

EC holds a PhD in Nursing and is Deputy Principal of Kamuzu College of Nursing, Malawi.

AM holds a PhD in Nursing and is Principal of Kamuzu College of Nursing, Malawi.

JØO holds a PhD, MD, Specialist in Gynecology / Obstetrics and is a Professor of International Health, University of Tromsø, Norway.

GB holds a PhD MD, Specialist in medical microbiology and immunology and is a Professor in community medicine, Institute of General Practice and Community Medicine, Faculty of Medicine, University of Oslo.

## References

[B1] National Statistical Office (NSO), ICF Macro National Statistical Office (NSO) ICF Macro: Malawi Demographic and Health Survey 2010 2011 Zomba, Malawi and Calverton, Maryland USA: NSO and ICF Macro

[B2] Ahman E Zupan J Neonatal and perinatal mortality: country, regional and global estimates 2004 2007 Geneva 27, Switzerland: World Health Organisation, Department of Making Pregnancy Safer

[B3] Ministry of Health Road Map for accelerating the reduction of maternal and neonatal mortality and morbidity in Malawi 2007 Lilongwe, Malawi: Ministry of Health

[B4] National Ministry of Health National Reproductive Health Standards 2008 Lilongwe, Malawi: Ministry of Health

[B5] Hulton LA Matthews Z Stones RW Applying a framework for assessing the quality of maternal health services in urban India Soc Sci Med 2007 64 2083 2095 10.1016/j.socscimed.2007.01.019 17374551

[B6] Pittrof R Campbell OM Filippi VG What is quality in maternity care? An international perspective Acta Obstet Gynecol Scand 2002 81 277 283 10.1034/j.1600-0412.2002.810401.x 11952455

[B7] Ministry of Health Malawi National reproductive Health Service Delivery Guidelines 2007 Lilongwe, Malawi: Ministry of Health

[B8] Hulton LA Matthews Z Stones RW A framework for the evaluation of quality of care in maternity services 2000 Highfield, Southampton: University of Southampton

[B9] National Statistical Office (NSO), ORC Macro Malawi Demographic Health Survey 2004 2005 Zomba, Malawi, Calverton, Maryland USA: NSO and ORC Macro

[B10] Lawn JE Lee AC Kinney M Sibley L Carlo WA Paul VK Two million intrapartum-related stillbirths and neonatal deaths: where, why, and what can be done? Int J Gynaecol Obstet 2009 107 Suppl 1 S5 S18 S19 19815202 10.1016/j.ijgo.2009.07.016

[B11] Wild K Barclay L Kelly P Martins N Birth choices in Timor-Leste: a framework for understanding the use of maternal health services in low resource settings Soc Sci Med 2010 71 2038 2045 10.1016/j.socscimed.2010.09.012 20971540

[B12] Otis KE Brett JA Barriers to hospital births: why do many Bolivian women give birth at home? Rev Panam Salud Publica 2008 24 46 53 10.1590/S1020-49892008000700006 18764994

[B13] Rani M Bonu S Harvey S Differentials in the quality of antenatal care in India Int J Qual Health Care 2008 20 62 71 18024998 10.1093/intqhc/mzm052

[B14] Mathole T Lindmark G Majoko F Ahlberg BM A qualitative study of women's perspectives of antenatal care in a rural area of Zimbabwe Midwifery 2004 20 122 132 10.1016/j.midw.2003.10.003 15177855

[B15] Moore BM Alex-Hart BA George IO Utilization of health care services by pregnant mothers during delivery: a community based study in Nigeria East Afr J Public Health 2011 8 49 51 22066284

[B16] Creel LC Sass JV Yinger NV Client-Centered Quality: Clients’ Perspectives and Barriers to Receiving Care 2002 Washington, DC: 20009, Population Reference Bureau http://www.popcouncil.org/pdfs/frontiers/QOC/QOC-clients.pdf

[B17] Harrison S Buettner PG MacLennan R Why do mothers still sun their infants? J Paediatr Child Health 1999 35 296 299 10.1046/j.1440-1754.1999.00362.x 10404454

[B18] Yi CH African American Women and Prenatal Care: Effect of Patient-Provider Interaction 2010 Ann Arbor, Michigan 48109: PhD thesis. University of Michigan, School of Nursing

[B19] Harrison S Buttner P Nowak M Maternal beliefs about the reputed therapeutic uses of sun exposure in infancy and the postpartum period Australian Midwifery 2005 18 22 28

[B20] Fabian HM Radestad IJ Waldenstrom U Childbirth and parenthood education classes in Sweden, Australia Acta Obstet Gynecol Scand 2005 84 436 443 15842207 10.1111/j.0001-6349.2005.00732.x

[B21] Schmied V Myors K Wills J Cooke M Preparing expectant couples for new-parent experiences: a comparison of two models of antenatal education J Perinat Educ 2002 11 20 27 17273305 10.1624/105812402X88803 PMC1599797

[B22] USAID, ACCESS Focused Antenatal Care: Providing integrated, individualized care during pregnancy 2007 USAID, ACCESS http://www.hivandsrh.org/es/popline/focused-antenatal-care-providing-integrated-individualized-care-during-pregnancy

[B23] Ministry of Health Participants’ manual in integrated maternal and neonatal care 2009 Lilongwe, Malawi: Reproductive Health Unit, Ministry of Health

[B24] Seljeskog L Sundby J Chimango J Factors influencing women's choice of place of delivery in rural Malawi–an explorative study Afr J Reprod Health 2006 10 66 75 10.2307/30032472 17518132

[B25] National Statistical Office 2008 Population and Housing Census 2008 Zomba, Malawi: National Statistical Office

[B26] National Statistical Office, United Nations Children's Fund Malawi Multiple Indicator Cluster Survey 2006 2008 Lilongwe, Malawi: National Statistical Office and UNICEF

[B27] Speziale HJS Carpenter DR Qualitative Research in Nursing: advancing the humanistic imperative 2007 Philadelphia: Lippincott Williams & Wilkins

[B28] Braun V Clarke V Using thematic analysis in psychology Qualitative Research in Psychology,Volume 3 2006 2 Bristol: University of the West of England 77 101 10.1191/1478088706qp063oa

[B29] Kvale S Rinkmann S Interviews: Learning the Craft of Qualitative Research Interviewing 2009 2 California: Sage Publications Inc

[B30] Shafiei T Small R McLachlan H Women’s views and experiences of maternity care: a study of immigrant Afghan women in Melbourne, Australia Midwifery 2012 28 198 203 10.1016/j.midw.2011.02.008 21458892

[B31] Bazant ES Koenig MA Women’s satisfaction with delivery care in Nairobi’s informal settlements Int J Qual Health Care 2009 21 79 86 10.1093/intqhc/mzn058 19208648

[B32] Goberna-Tricas J Banus-Gimenez MR Palacio-Tauste A Linares-Sancho S Satisfaction with pregnancy and birth services: the quality of maternity care services as experienced by women Midwifery 2011 27 e231 e237 10.1016/j.midw.2010.10.004 21145632

[B33] Yakong VN Rush KL Bassett-Smith J Bottorff JL Robinson C Women’s experiences of seeking reproductive health care in rural Ghana: challenges for maternal health service utilization J Adv Nurs 2010 66 2431 2441 10.1111/j.1365-2648.2010.05404.x 20735507

[B34] Delvaux T Ake-Tano O Gohou-Kouassi V Bosso P Collin S Ronsmans C Quality of normal delivery care in cote d’ivoire Afr J Reprod Health 2007 11 22 32 10.2307/30032485 17982945

[B35] Muula AS Will health rights solve Malawi's health problems? Croat Med J 2005 46 853 859 16158484

[B36] Changole J Bandawe C Makanani B Nkanaunena K Taulo F Malunga E Patients’ satisfaction with reproductive health services at Gogo Chatinkha Maternity Unit, Queen Elizabeth Central Hospital, Blantyre, Malawi Malawi Med J 2010 22 5 9 21618840 10.4314/mmj.v22i1.55899 PMC3345683

[B37] Lungu F Malata A Chirwa E Mbendera I Quality assessment of focused antenatal care services in Malawi Afr J Midwifery & Womens Health 2011 5 169 175

[B38] Nikiema B Beninguisse G Haggerty JL Providing information on pregnancy complications during antenatal visits: unmet educational needs in sub-Saharan Africa Health Policy Plan 2009 24 367 376 10.1093/heapol/czp017 19401360

[B39] Bluff R Holloway I ‘They know best’: women's perceptions of midwifery care during labour and childbirth Midwifery 1994 10 157 164 10.1016/0266-6138(94)90046-9 7815955

[B40] Campero L Garcia C Diaz C Ortiz O Reynoso S Langer A “Alone, I wouldn’t have known what to do”: a qualitative study on social support during labor and delivery in Mexico Soc Sci Med 1998 47 395 403 10.1016/S0277-9536(98)00077-X 9681909

[B41] Senarath U Fernando DN Rodrigo I Factors determining client satisfaction with hospital-based perinatal care in Sri Lanka Trop Med Int Health 2006 11 1442 1451 10.1111/j.1365-3156.2006.01698.x 16930267

[B42] Tayelgn A Zegeye DT Kebede Y Mothers’ satisfaction with referral hospital delivery service in Amhara Region, Ethiopia BMC Pregnancy Childbirth 2011 11 78 10.1186/1471-2393-11-78 22023913 PMC3254067

[B43] Dongre AR Deshmukh PR Garg BS A community based approach to improve health care seeking for newborn danger signs in rural Wardha, India Indian J Pediatr 2009 76 45 50 10.1007/s12098-009-0028-y 19391002

[B44] Fujino Y Sasaki S Igarashi K Tanabe N Muleya CM Tambatamba B Improvement in mothers' immediate care-seeking behaviors for children's danger signs through a community-based intervention in Lusaka, Zambia Tohoku J Exp Med 2009 217 73 85 10.1620/tjem.217.73 19155611

[B45] Sasaki S Fujino Y Igarashi K Tanabe N Muleya CM Suzuki H Access to a health facility and care-seeking for danger signs in children: before and after a community-based intervention in Lusaka, Zambia Trop Med Int Health 2010 15 312 320 10.1111/j.1365-3156.2009.02460.x 20070629

[B46] Ministry of Health Clinical Management of HIV in Children and Adults 2011 Lilongwe, Malawi: Ministry of Health

[B47] Chanza D Chirwa E Maluwa A Malata A Masache G Factors affecting the choice for home deliveries in Malawi Afr J Midwifery & Womens Health 2012 6 125 130

[B48] Small R Yelland J Lumley J Brown S Liamputtong P Immigrant women’s views about care during labor and birth: an Australian study of Vietnamese, Turkish, and Filipino women Birth 2002 29 266 277 10.1046/j.1523-536X.2002.00201.x 12431266

[B49] D’Ambruoso L Abbey M Hussein J Please understand when I cry out in pain: women’s accounts of maternity services during labour and delivery in Ghana BMC Public Health 2005 5 140 10.1186/1471-2458-5-140 16372911 PMC1343547

[B50] Mrisho M Schellenberg JA Mushi AK Obrist B Mshinda H Tanner M Factors affecting home delivery in rural Tanzania Trop Med Int Health 2007 12 862 872 10.1111/j.1365-3156.2007.01855.x 17596254

[B51] Kongnyuy EJ Hofman J Mlava G Mhango C van den Broek N Availability, utilisation and quality of basic and comprehensive emergency obstetric care services in Malawi Matern Child Health J 2009 13 687 694 10.1007/s10995-008-0380-y 18581221

[B52] Foster J Burgos R Tejada C Caceres R Altamonte AT Perez LJ A community-based participatory research approach to explore community perceptions of the quality of maternal-newborn health services in the Dominican Republic Midwifery 2010 26 504 511 10.1016/j.midw.2010.06.001 20692744 PMC2946473

[B53] Grossmann-Kendall F Filippi V De KM Kanhonou L Giving birth in maternity hospitals in Benin: testimonies of women Reprod Health Matters 2001 9 90 98 10.1016/S0968-8080(01)90095-3 11765405

[B54] Oyo-Ita AE Etuk SJ Ikpeme BM Ameh SS Nsan EN Patients’ perception of obstetric practice in Calabar, Nigeria Niger J Clin Pract 2007 10 224 228 18072450

[B55] Gao Y Barclay L Kildea S Hao M Belton S Barriers to increasing hospital birth rates in rural Shanxi Province, China Reprod Health Matters 2010 18 35 45 10.1016/S0968-8080(10)36523-2 21111349

[B56] Ratsma YEC Lungu K Hofman JJ White SA Why more mothers die: confidential equiries into institutional maternal deaths in the Southern Region of Malawi, 2001 Malawi Med J 2005 17 75 80 10.4314/mmj.v17i3.10882 PMC3345509 27529003

[B57] Chodzaza E Bultemeier K Service providers’ perception of the quality of emergency obsteric care provided and factors indentified which affect the provision of quality care Malawi Med J 2010 22 104 111 21977830 10.4314/mmj.v22i4.63946 PMC3345772

[B58] Zimba E Kinney MV Kachale F Waltensperger KZ Blencowe H Colbourn T Newborn survival in Malawi: a decade of change and future implications Health Policy Plan 2012 27 Suppl 3 iii88 iii103 10.1093/heapol/czs043 22692419

[B59] Kinney MV Kerber KJ Black RE Cohen B Nkrumah F Coovadia H Sub-Saharan Africa’s mothers, newborns, and children: where and why do they die? PLoS Med 2010 7 e1000294 10.1371/journal.pmed.1000294 20574524 PMC2888581

[B60] Sines E Syed U Wall S Worley H Postnatal Care: A critical opportunity to save mothers and newborns 2007 Save the children, Population Reference Bureau, Washington, DC 20036 USA, Washington, DC 20009 USA http://www.prb.org/pdf07/snl_pncbrieffinal.pdf

[B61] Lawn JE Cousens S Zupan J 4 million neonatal deaths: when? Where? Why? Lancet 2005 365 891 900 10.1016/S0140-6736(05)71048-5 15752534

